# Supplementary material for: Structural and Functional Characterization of a Testicular Long Non-coding RNA (4930463O16Rik) Identified in the Meiotic Arrest of the Mouse Topaz1–/– Testes
Source: Front Cell Dev Biol. 2021 Jul 1;9:700290. doi: 10.3389/fcell.2021.700290 (PMC8281061; doi:10.3389/fcell.2021.700290)
Supplement: Supplementary Figure 1 — ISH control probe expression in WT 2-month-old testes. (A) Probe targeting the bacterial gene dapB (dapB, Bacillus subtilis dihydrodipicolinate reductase) was used as a negative control; (B,C) are examples using positive control probes (PPIB, Mus musculus peptidylprolyl isomerase B and UBC, Homo sapiens ubiquitin C). Probes for housekeeping genes (PPIB and UBC, respectively weakly and highly expressed genes) were used as positive controls (brown dots). [file Data_Sheet_1.PDF]

**Supplementary Figures:**

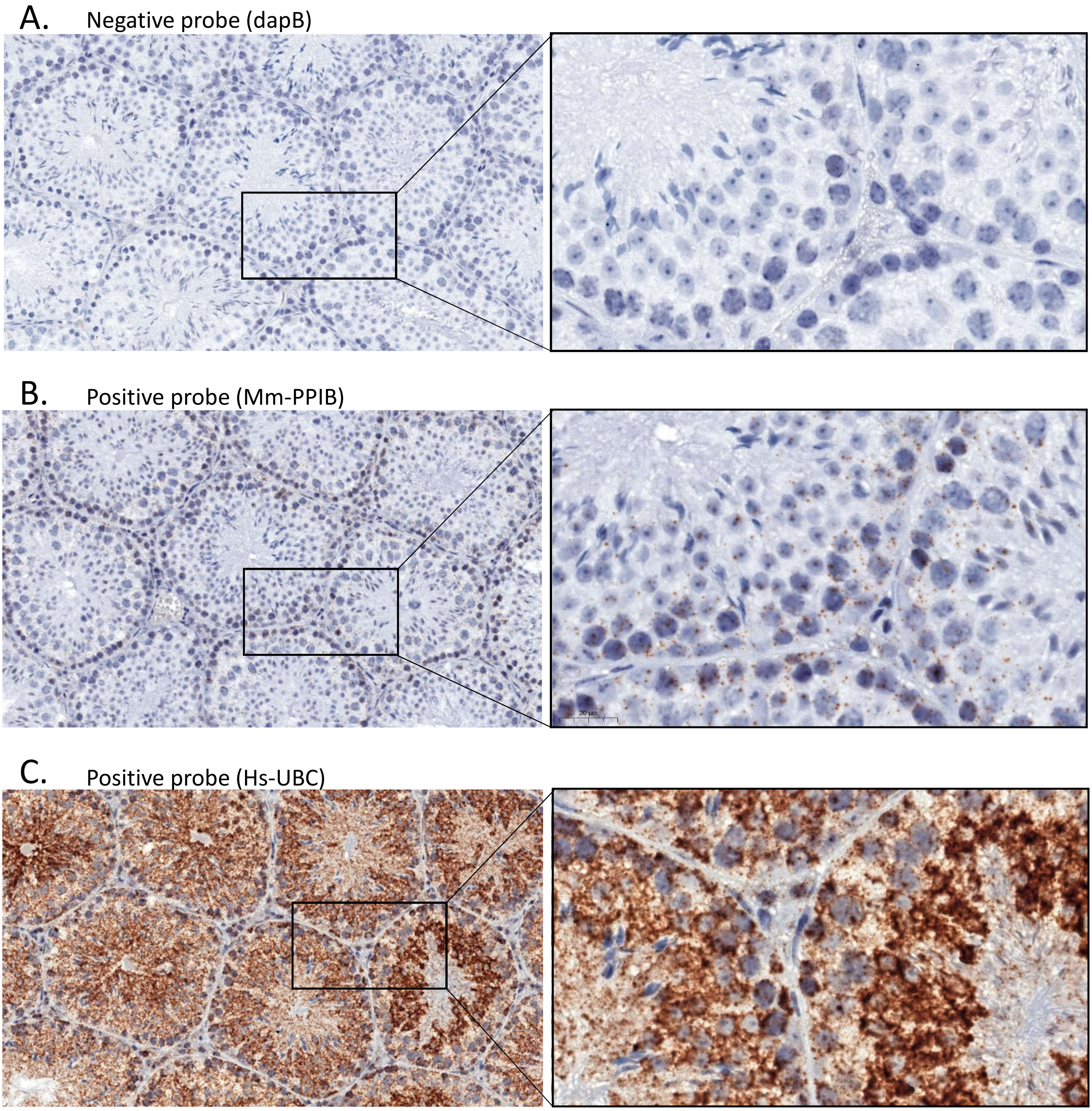


**Supplementary Figure 1.** I*SH* control probe expression in WT 2-month-old testes. (**A**) Probe targeting the bacterial gene dapB (dapB, Bacillus subtilis dihydrodipicolinate reductase) was used as a negative control; (**B** and **C**) are examples using positive control probes (PPIB, Mus musculus peptidylprolyl isomerase B and UBC, Homo sapiens ubiquitin C). Probes for housekeeping genes (PPIB and UBC, respectively weakly and highly expressed genes) were used as positive controls (brown dots).

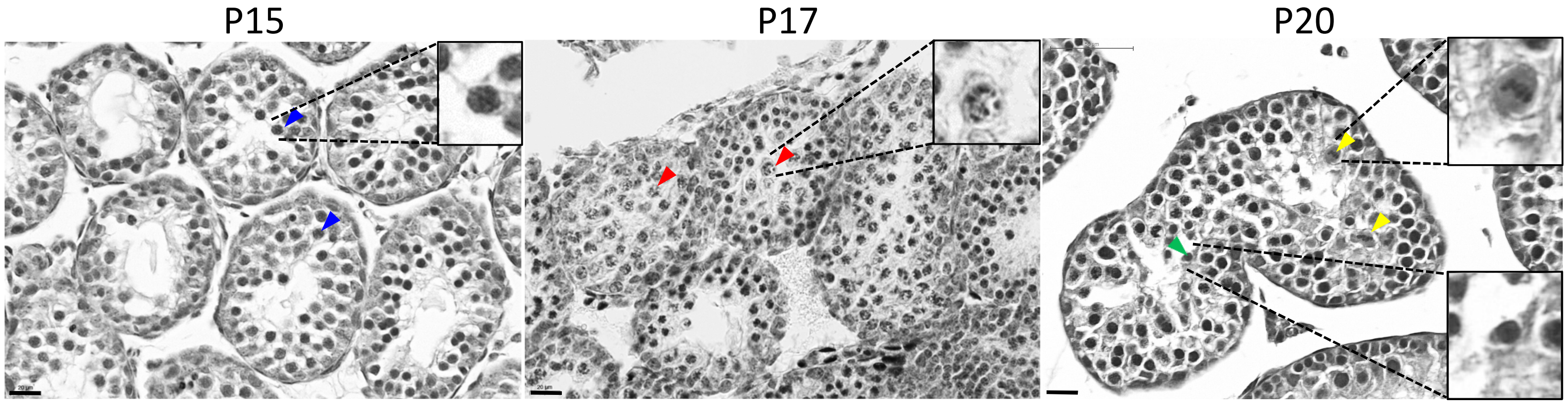


**Supplementary Figure 2:** Hematoxylin and eosin staining of wild-type testes **(A)** at P15; **(B)** P17 and **(C)** P20 in mice (C57/Bl6). At P15, seminiferous tubules contain spermatocytes that have advanced to early and mid-pachynema (blue arrowhead); at P17, early-mid diplotene cells (red arrowhead) appear. At P20, according to the seminiferous epithelium stages, metaphase I plate in spermatocytes are visible (yellow arrowhead) or first round spermatids (green arrowhead) resulting from the first meiotic division. Scale bar=20 μm

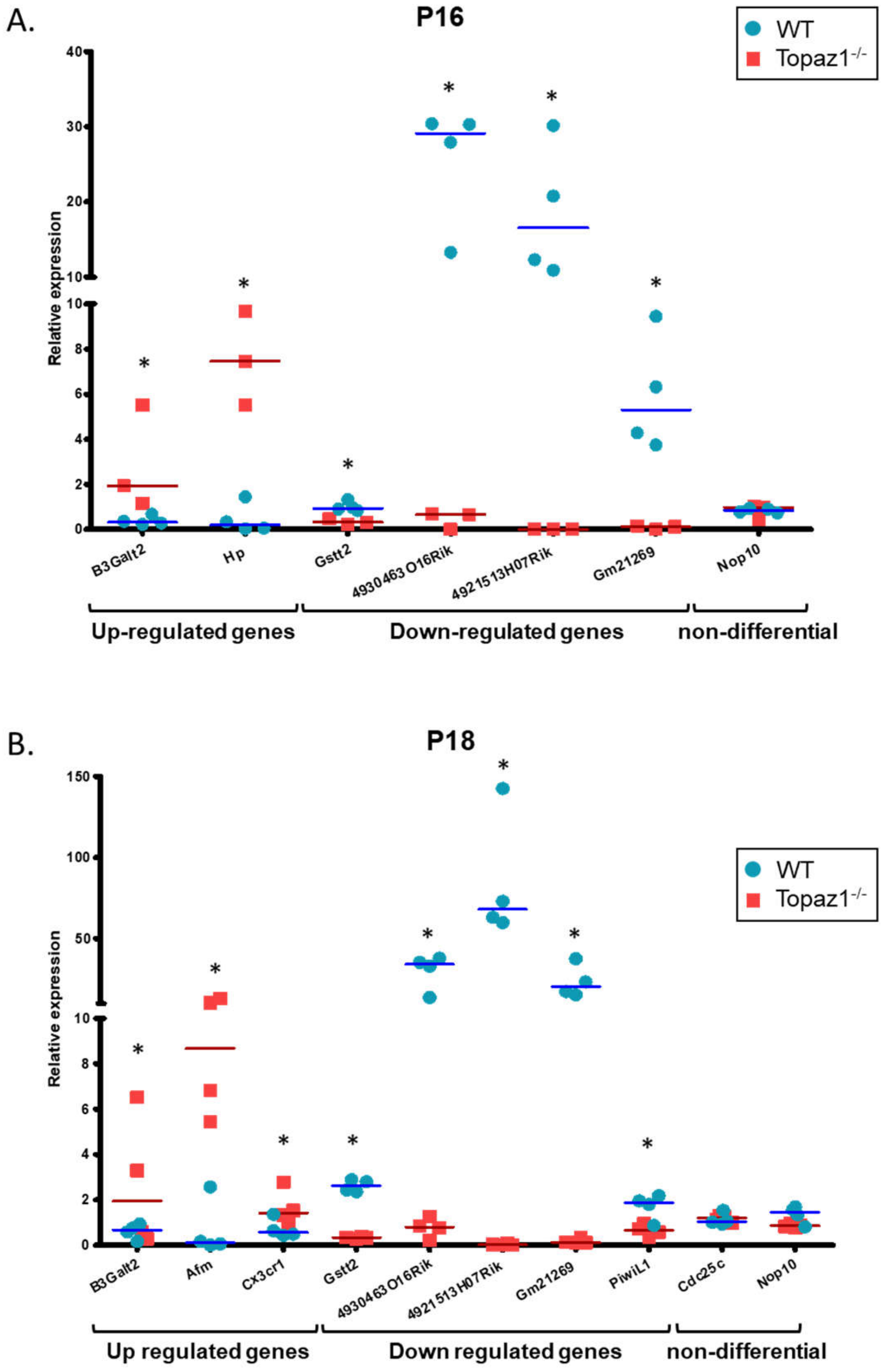


**Supplementary Figure 3.** Validation of several DEGs by RT-qPCR (RNA-seq *Topaz1*$^{-/-}$ *vs* WT testes). Validation of several differentially expressed up- or down-regulated genes and of non-DEGs from RNA-seq analysis by qRT-PCR from P16 **(A)** or P18 **(B)** mouse testis RNAs. The lines represent the median of each genotype (blue: WT; red: *Topaz1*$^{-/-}$). A Kruskal-Wallis statistical test was performed (*p<0.05).

## Dynamic expression of *4930463O16Rik*

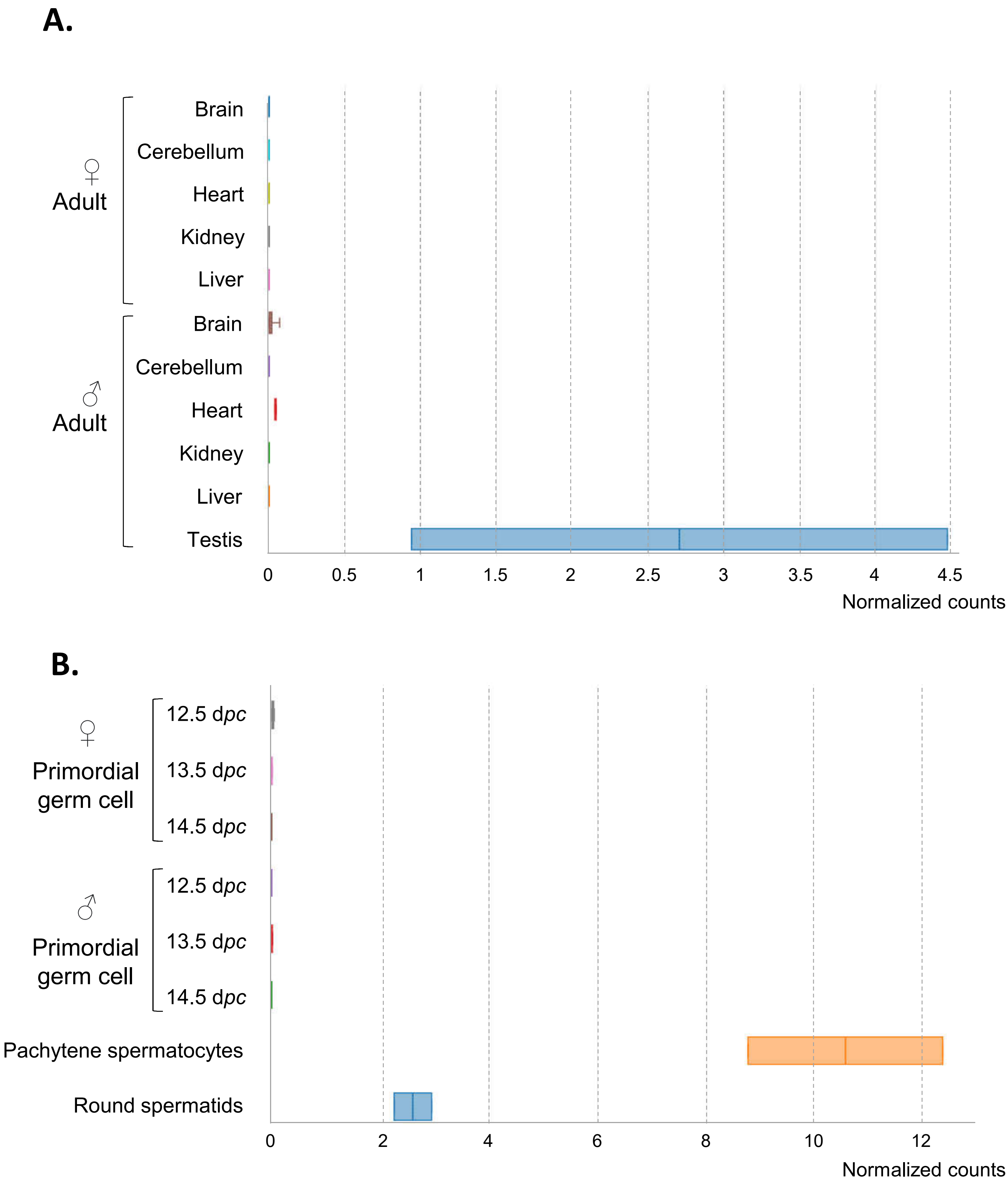


**Supplementary Figure 4.** Reprogenomic data on the dynamic expression of *4930463O16Rik*. The dynamic expression of *4930463O16Rik* in five different tissues from male and female adult mice **(A)**, and in embryonic primordial germ cells and adult male germ cells **(B)**. *4930463O16Rik* is expressed in testes in germ cells during post-natal life. The strongest dynamic expression is found in pachytene spermatocytes. The X-axes represented normalized counts.

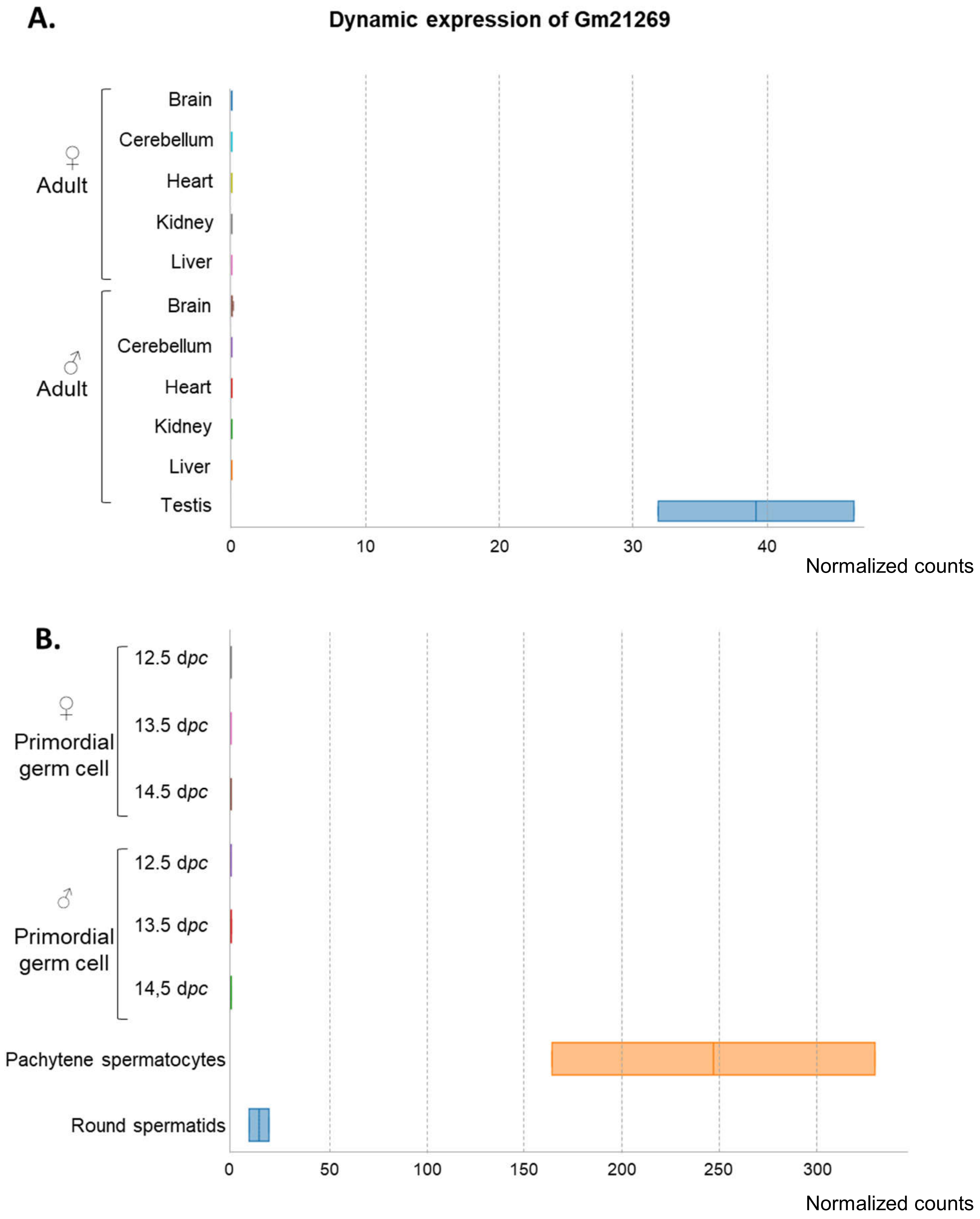


**Supplementary Figure 5.** Reprogenomic data on the dynamic expression of *Gm21269*. The dynamic expression of *Gm21269* in five different tissues from male and female adult mice **(A)**, in embryonic primordial germ cells and adult male germ cells **(B)**. *Gm21269* is expressed in testes in germ cells during post-natal life. The strongest dynamic expression is found in pachytene spermatocytes. The X-axes represented normalized counts.

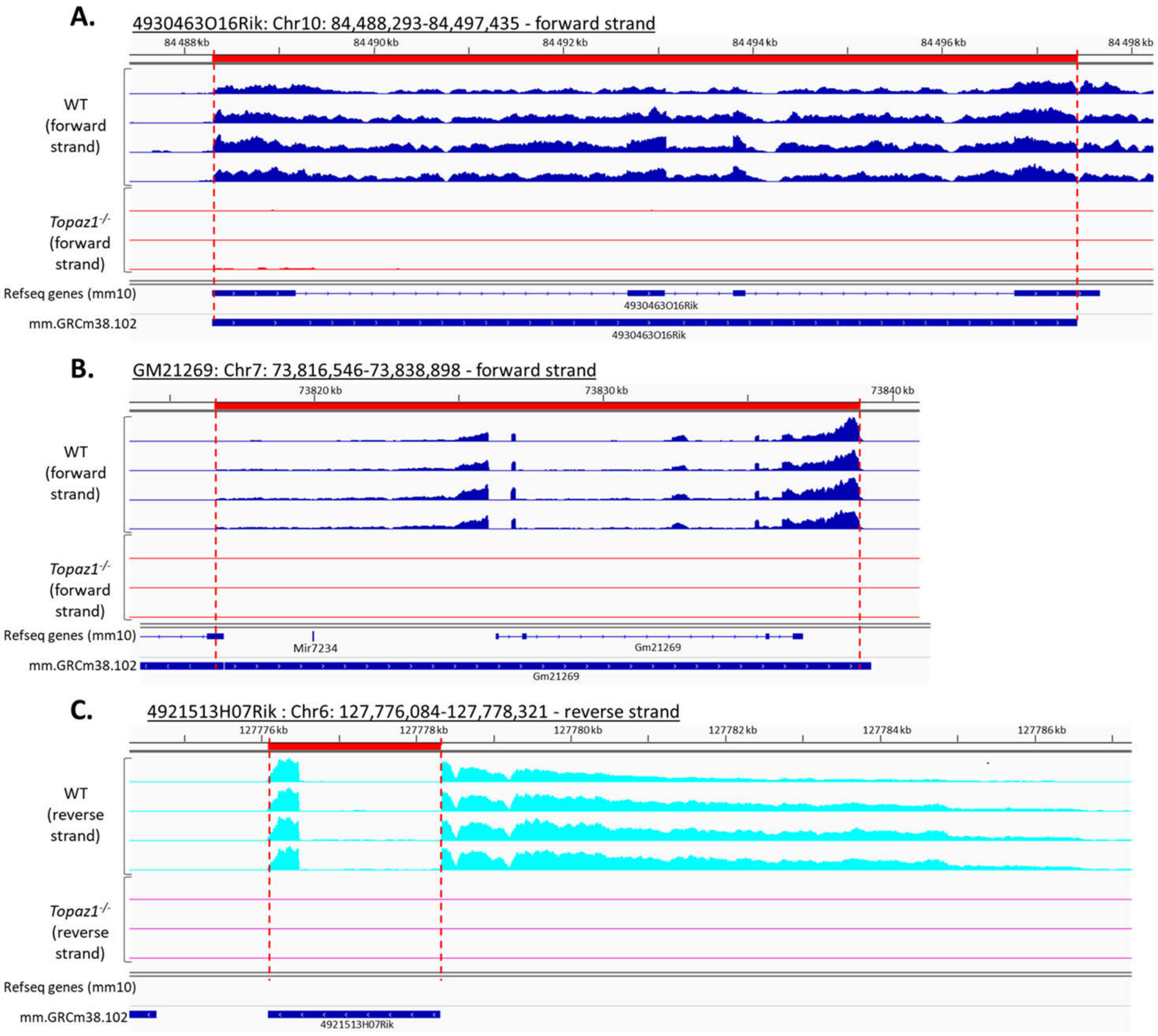


**Supplementary Figure 6.** IGV representation of P18-testis RNA-seq. Expression of *4930463O16Rik* **(A)**, *Gm21269* **(B)** and *4921513H07Rik* **(C)** from BigWig files of strand-specific RNA-seq data. The first four tracks represent transcripts of WT testes at P18; the next three tracks represent transcripts of *Topaz1*$^{-/-}$ testes at the same developmental stage. Representations of the genes (from mm10 or GRCm38) are shown at the bottom of each graph. A representation of the size of *4930463O16Rik* **(A)**, *Gm21269* **(B)** and *492151H07Rik* **(C)** transcripts (red) from Ensembl data (GRCm38) is shown at the top. *4930463O16Rik* and *4921513H07Rik* gene transcriptions overlap in 3' or 5', respectively.

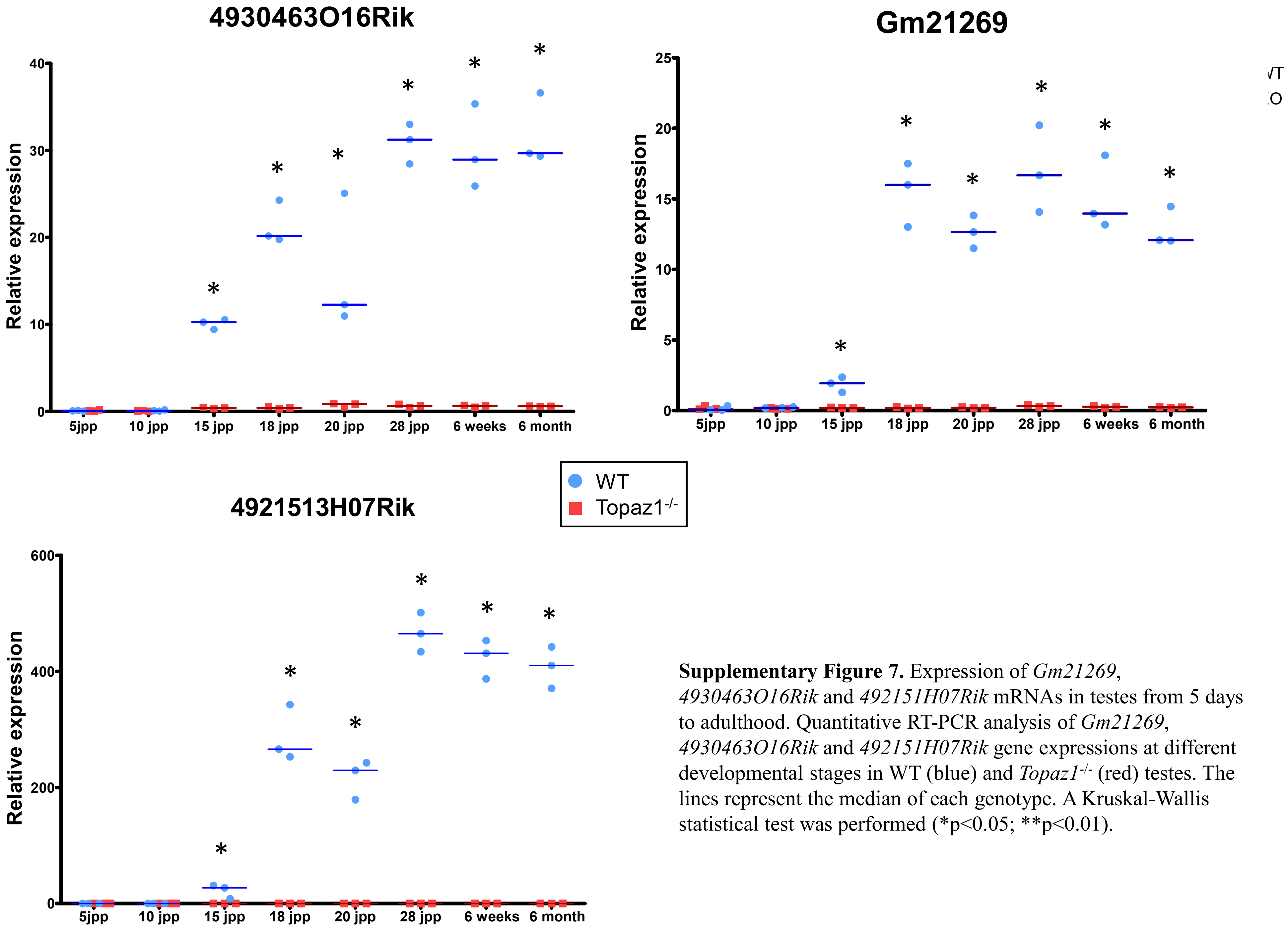


**Supplementary Figure 7.** Expression of *Gm21269*, *4930463O16Rik* and *492151H07Rik* mRNAs in testes from 5 days to adulthood. Quantitative RT-PCR analysis of *Gm21269*, *4930463O16Rik* and *492151H07Rik* gene expressions at different developmental stages in WT (blue) and *Topaz1*$^{-/-}$ (red) testes. The lines represent the median of each genotype. A Kruskal-Wallis statistical test was performed (*p<0.05; **p<0.01).

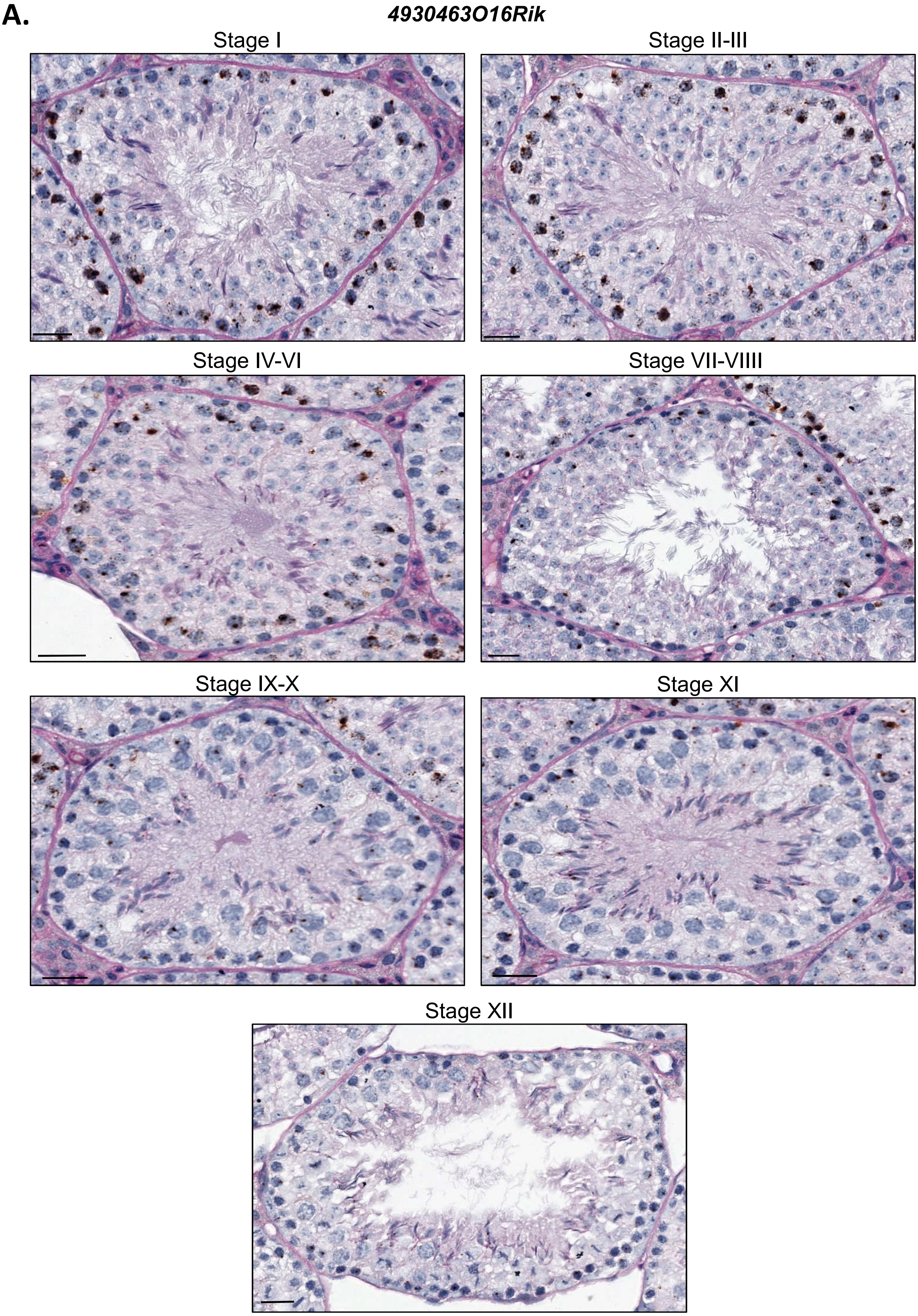

A.
4930463O16Rik
Stage I
Stage II-III
Stage IV-VI
Stage VII-VIIII
Stage IX-X
Stage XI
Stage XII

**B.** *Gm21269*

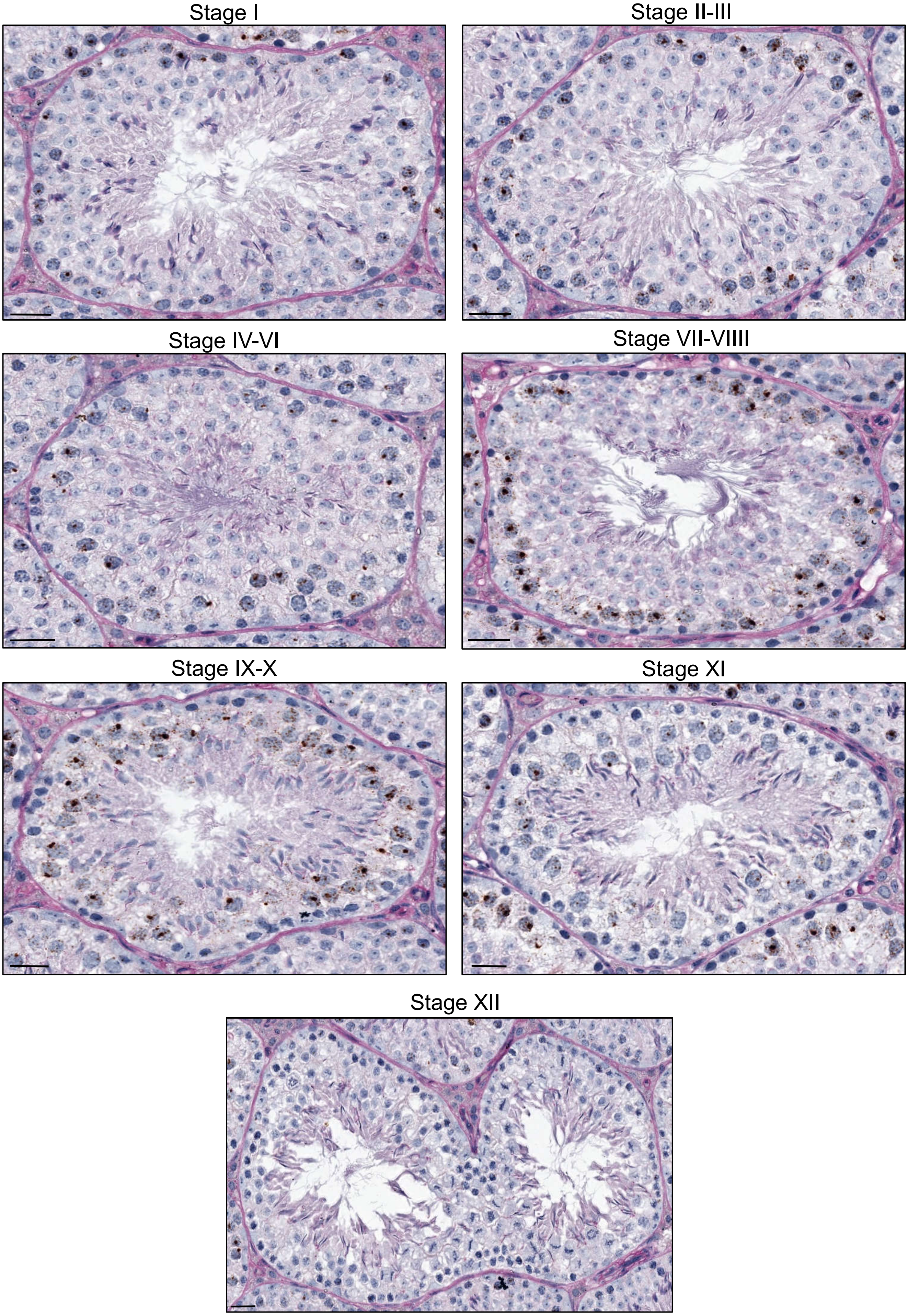

Stage I
Stage II-III
Stage IV-VI
Stage VII-VIIII
Stage IX-X
Stage XI
Stage XII

**C.** ***4921513H07Rik***

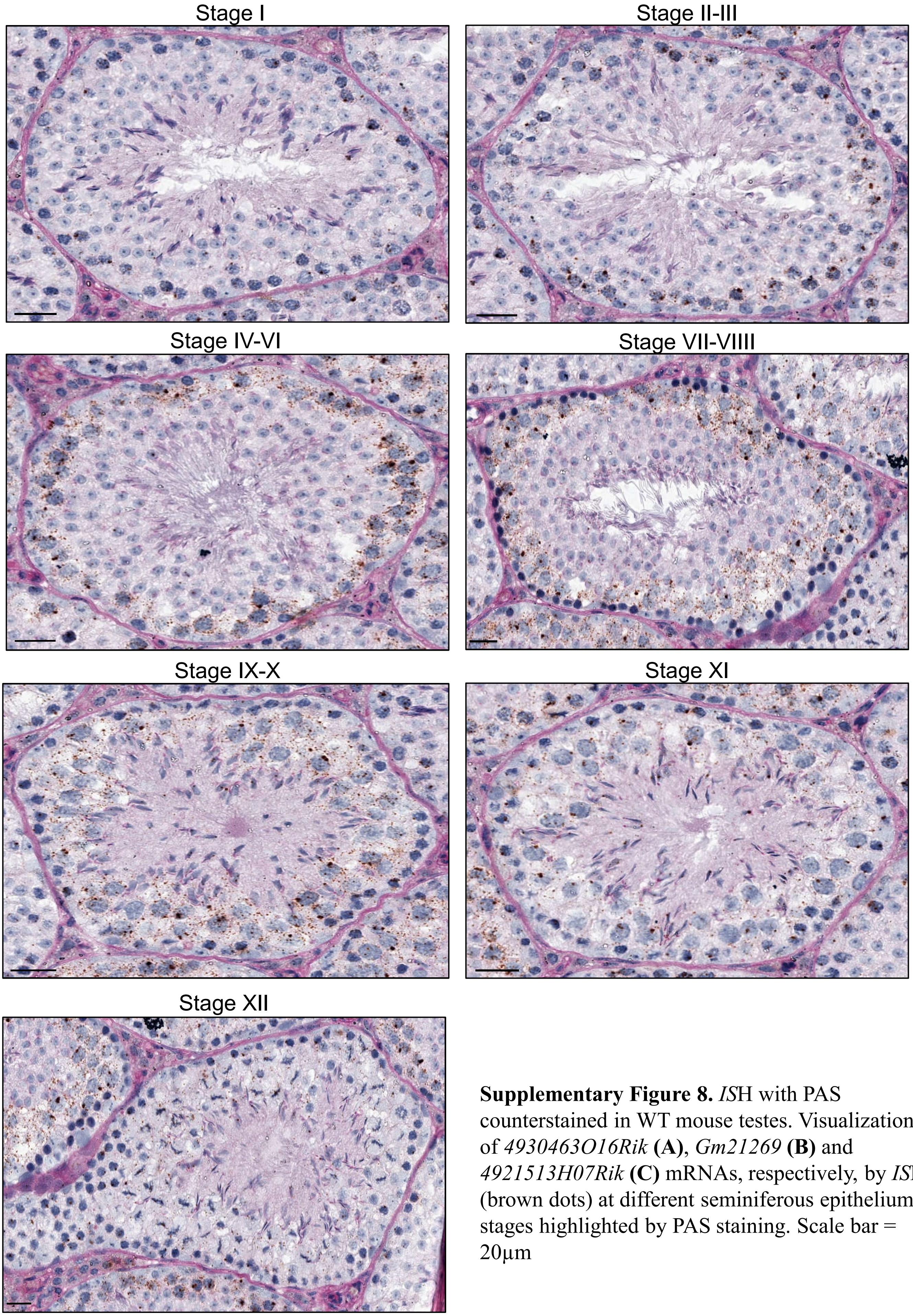


**Supplementary Figure 8.** *IS*H with PAS counterstained in WT mouse testes. Visualization of *4930463O16Rik* **(A)**, *Gm21269* **(B)** and *4921513H07Rik* **(C)** mRNAs, respectively, by *IS*H (brown dots) at different seminiferous epithelium stages highlighted by PAS staining. Scale bar = 20μm

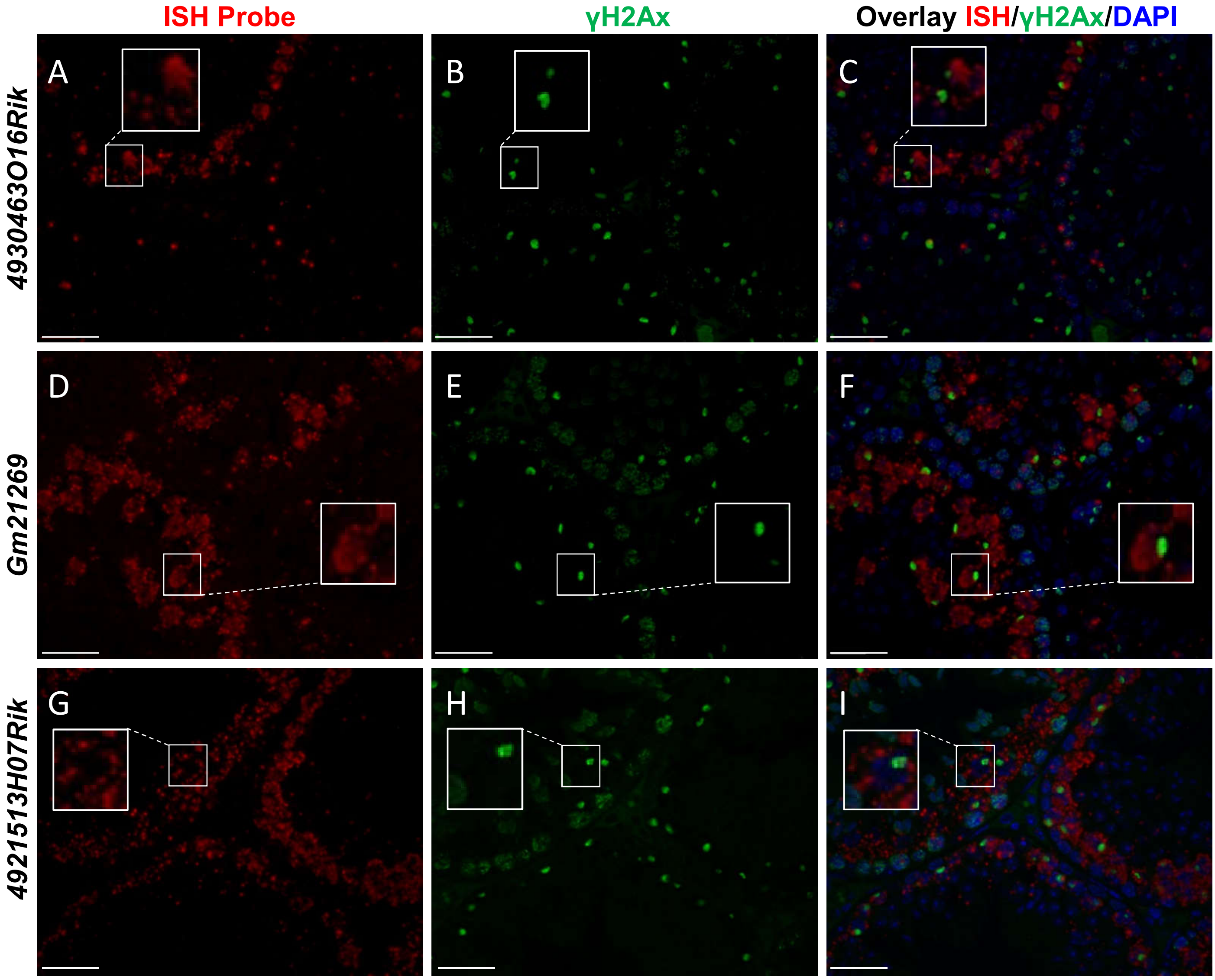


**Supplementary Figure 9.** LncRNA cellular localizations in testes from two month-old WT mice. *IS*H using **(A)** *4930463O16Rik*, **(D)** *Gm21269* and **(G)** *4921513H07Rik* probes (red). **(B-E-H)** Immunofluorescence staining with γH2Ax antibody was performed at the same stage of seminiferous epithelium to identify male germ cells (green). **(C-F-I)** DAPI (blue), visualizing nuclear chromosomes, was merged with *IS*H (green) and IF (red) signals. Zooms in white squares show spermatocytes during prophase I. No colocation between the sex body (γH2Ax) and lncRNAs (red) was evident. Scale bar = 20 µm.

**A.**

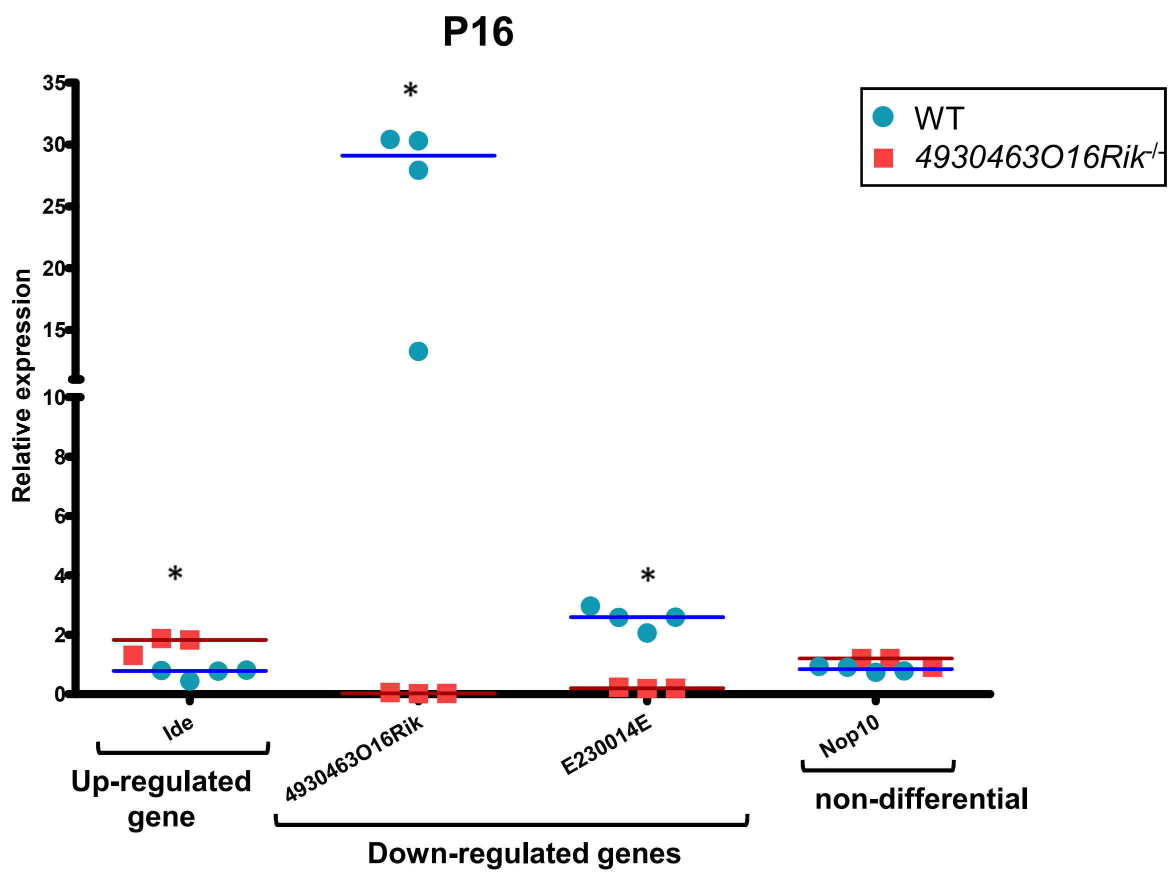


**B.**

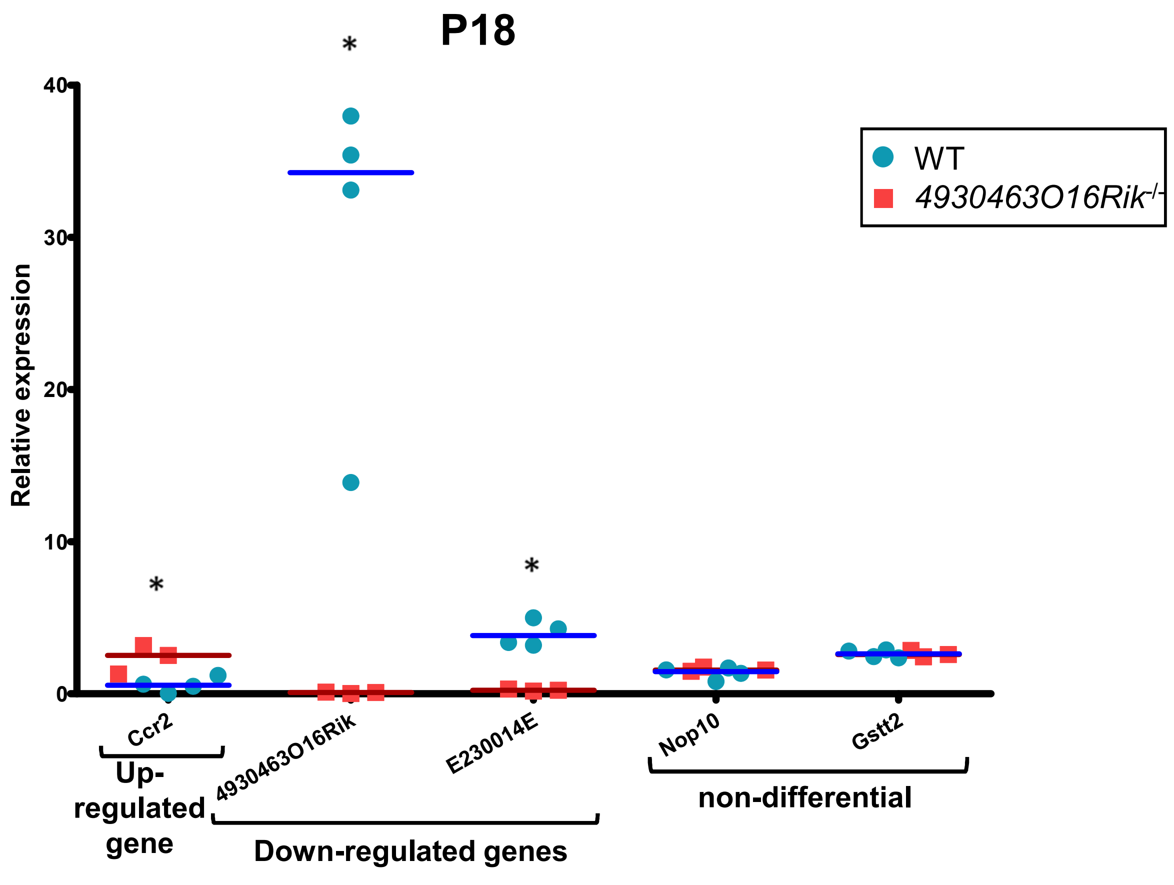


**Supplementary Figure 10.** Validation of several DEGs by RT-qPCR (RNA-seq *4930463O16Rik*$^{-/-}$ *vs* WT testes). Validation of several differentially expressed up- or down-regulated genes and of non-DEGs of RNA-seq analysis by RT-qPCR from P16 **(A)** or P18 **(B)** mouse testis RNAs. The lines represent the median of each genotype (blue: WT; red: 4930463O16Rik$^{-/-}$). A Kruskal-Wallis statistical test was performed (*$p<0.05$).
